# Visualization as a stimulus domain for vision science

**DOI:** 10.1167/jov.21.8.3

**Published:** 2021-08-02

**Authors:** Ronald A. Rensink

**Affiliations:** 1 Departments of Computer Science and Psychology, University of British Columbia, Vancouver, Canada

**Keywords:** visual cognition, information visualization, data visualization, perceptual organization, visual system, computational theory

## Abstract

Traditionally, vision science and information/data visualization have interacted by using knowledge of human vision to help design effective displays. It is argued here, however, that this interaction can also go in the opposite direction: the investigation of successful visualizations can lead to the discovery of interesting new issues and phenomena in visual perception. Various studies are reviewed showing how this has been done for two areas of visualization, namely, graphical representations and interaction, which lend themselves to work on visual processing and the control of visual operations, respectively. The results of these studies have provided new insights into aspects of vision such as grouping, attentional selection and the sequencing of visual operations. More generally yet, such results support the view that the perception of visualizations can be a useful domain for exploring the nature of visual cognition, inspiring new kinds of questions as well as casting new light on the limits to which information can be conveyed visually.

## Introduction

One of the more striking characteristics of modern society is its ever-increasing use of data, in both the number of sectors involved (e.g., health, industry, environment) as well as the amount of information used in each. To enable the resulting large datasets to be efficiently analyzed, various approaches have been developed. Among the more prominent is *information (or data) visualization*,^1^ in which information is displayed in a graphical format that can be interactively controlled by a human analyst. The use of a visualization system can enable an analyst to obtain a quick overview of a dataset, examine individual items, and discover whatever structure may exist (e.g., trends, outliers, sudden changes). If the system is designed well, the analysis of a large dataset can be rapid, accurate, and precise. Such a system essentially amplifies the intelligence of the user by “using vision to think,” that is, remapping problems into a form that enables visual intelligence to be brought to bear (Card, Mackinlay, & Shneiderman, 1999). As such, there is considerable potential for a productive interaction between the study of visualization and the study of visual perception.

In the past, this interaction generally took the form of using knowledge of perception to help design effective visualizations (e.g., MacEachren, 1995; Spence, 2014; Ware, 2012). However, another approach has recently emerged that develops connections in the opposite direction: using experiments on visualizations to provide new insights into visual perception. This article describes how this newer approach operates and what it has accomplished to date, reviewing various representative studies. The limitations of this approach are also discussed, as well as possible directions for future research.

## Basics

### Visualization

**Figure 1.**
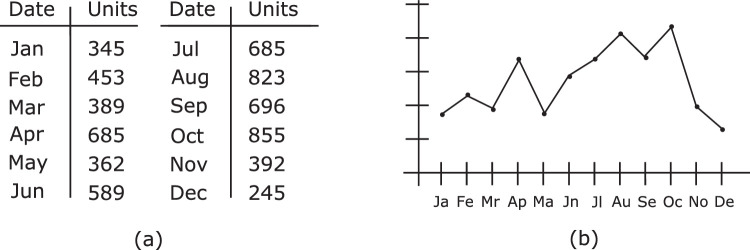
Different representations of an example dataset. (a) Tabular form. (b) Line graph. In general, both trends and outliers are more easily and quickly seen in line graphs.

Visualization—in the broad sense considered here—can be thought of as *the transformation of a dataset into graphical form, so as to best employ the visual intelligence of a human analyst* (cf. Card et al., 1999). For example, the sales records for a company over the course of a year could be displayed as a numerical table or a line graph (Figure 1). Although both representations display the same information, a line graph enables the viewer to easily perceive things such as sales trends and peak sales times. A table can, of course, enable exact values to be displayed, but using it to perceive structure in the data would usually require much more time and effort, especially as the size of the dataset grows. For many analytic tasks, then, a representation like a line graph can be far more useful than simple text, motivating the widespread use of visualization in analysis.

Central to the process of visualization is a *visualization pipeline* that maps the information in an abstract dataset to a *graphical representation* on a spatial medium of some kind, such as a computer display (Card et al., 1999; Munzner, 2014; Spence, 2014). When viewed, the graphical representation gives rise to a *visual representation* in the analyst (user), which then serves as the basis for further perceptual and cognitive processing. In an interactive system the user can control what is displayed at any moment—for example., drill down to a particular item in the dataset, zoom out to get a global view of general trends, or filter out irrelevant items to find patterns in the remainder. The effectiveness of a given visualization system for a particular task, therefore, depends on the extent to which these actions enable the appropriate mechanisms—both human and machine—to be brought to bear at the appropriate time.

### Connections from vision science

Historically, the most common way that vision science interacted with visualization was by using knowledge of perception to help with design (e.g., Few, 2013; MacEachren, 1995, Montello, 2002; Ware, 2008, 2012; Zacks & Franconeri, 2020). Issues here include the choice of features such as color, contrast, or orientation to best encode scalar values (e.g., temperature or pressure), or to best separate classes of data (e.g., cats and dogs) that are presented simultaneously (e.g., Brewer, 1999; Roth, 2017; Ware, 2012). Studies also examined the potential of processes such as texture perception (Hagh-Shenas, Interrante, & Park, 2006) and ensemble coding (Cui & Liu, 2021; Szafir, Haroz, Gleicher, & Franconeri, 2016). Knowledge of peripheral vision (e.g., the effects of crowding) has likewise been used to help increase the amount of information that could be picked up at any instant (Rosenholtz, 2011).

This approach has also been applied to higher level perceptual and cognitive processes. One example is the control of visual attention, which—if handled properly—can help a viewer to focus on the right information at the right time. A visualization system that has been designed to work well with attentional mechanisms could, for instance, use properties that minimize the chance of attention being inadvertently drawn away, use the grouping of items to reduce perceived clutter, and use cueing to actively direct attention (e.g., Healey & Enns, 2012; Rensink, 2011; Rosenholtz, Li, & Nakano, 2007; Wickens & McCarley, 2008).

A somewhat different set of connections involves the methodology for assessing how well a given visualization works. A “first wave” of experimental work measured accuracy—and occasionally, time needed—for various tasks in simple settings (e.g., Cleveland & McGill, 1984, Lewandowsky & Spence, 1989). However, it is increasingly being recognized that more could be done in this regard (Kosara & Haroz, 2018). Vision science can clearly help with this work by providing techniques to assess various aspects of behavior (Elliott, Nothelfer, Xiong, & Szafir, 2021; Rensink & Baldridge, 2010). For instance, just noticeable differences (JNDs) reflect the variability of an observer's percepts about a central tendency. Applied to the perception of correlation in scatterplots, for example, JNDs could show which of two designs has a better “resolution,” that is, better enable the user to notice differences between the structure of two datasets. In cartography, the use of JNDs (or equivalents) has helped to ensure that symbols always appear distinct (see Griffin, 2017; Griffin & Montello, 2015; MacEachren, 1995), and has helped assess how well a given map design supports inferences about spatial structure (Beecham et al., 2017).

### Connections *to* vision science

Although the value of contributions *from* vision science to visualization has long been recognized, awareness has recently emerged of connections in the opposite direction: from visualization *to* vision science. A key component of this “reverse” approach is a view of visualization systems not as tools for analyzing data, but as objects of scientific interest in their own right—a stimulus domain similar to, say, objects or natural scenes. The goal of such studies is to understand how a given visualization works—that is, to measure its performance under various conditions, and determine the perceptual mechanisms involved. The generality of the results obtained this way are usually greater than those obtained via traditional user studies, where a particular visualization is assessed only in terms of its suitability for a particular purpose (Kosara & Haroz, 2018). And by their very nature, such general results also have the potential to cast light on important aspects of our visual intelligence.

It might be argued that studies of this kind are inherently misguided, in that visualization systems are pure artifacts—in contrast with scene perception, say, human vision did not evolve to cope with line graphs or scatterplots. But just because a system is an artifact does not mean it is arbitrary. Most visualizations in popular use are the survivors of a considerable amount of competition (e.g., see Friendly & Denis, 2005). Consequently, even though we did not evolve to work with visualizations, they—or at least, the more effective ones—essentially evolved to work with us (Rensink, 2017). These survivors are therefore not arbitrary constructs, but are instead systems that embody considerable information about how we perceive and think. If this information can be extracted, it may have the potential to provide a fast track to understanding several of our perceptual and cognitive processes, and perhaps even provide new insights into the nature of the divide between perception and cognition themselves.

### Approach

The reverse approach to investigating visualizations considers these as objects of interest in their own right, and investigates them accordingly. Such work—done by researchers in both vision science and visualization—can be seen as a way to go beyond the “simple world” assumptions of traditional vision science, allowing us to explore aspects of real-world situations such as the perception of complex objects in heterogeneous backgrounds (Szafir, 2017). They can also inspire new kinds of questions, such as those about the degree (and nature) of the visual intelligence we have, and the extent to which our visual system is hardwired for the natural world.

Studies on visualization use a strategy common to much of science: focus on aspects of a system that are complex enough to be interesting, but simple enough to allow easy experimental manipulation. (A well-known example of this is the use of fruit flies in biological research.) In particular, this strategy contains five elements:

1. *Find* *or* *create a simple version of a commonly* *used visualization*. This version has a relatively simple design involving a minimal number of factors (in regards to colors, shapes, etc.). It should still, however, be complex enough to support a basic functionality.
2. *Select one of its functions* *or* *tasks*. Visualization systems are often capable of supporting several functions—for example, a scatterplot can enable both the estimation of correlation and the perception of data clusters. In the interests of simplicity, the experimental task should focus on just one of these.
3. *Measure performance under controlled conditions*. Using one or more forms of measurement, determine how performance is affected by easily controllable aspects of the visualization such as color, size, or timing. Properties of the data distributions used may also be of interest.
4. *Find regularities in this performance*. Ideally, behavior can be described by a relatively simple law. If not (as is often the case), describe regularities such as the factors that do or do not affect performance, and if possible, connect to known perceptual laws.
5. *Find explanations for these regularities*. As much as possible, explain behavior in terms of known perceptual mechanisms. If this cannot be done, posit new mechanisms compatible with what is known. And if this is not possible, at least attempt to draw some implications for known mechanism.

First-wave work on visualization typically had the first three of these. Newer work adds the latter two, enabling us to assess not only *how well* a visualization system works, but also *why*; the result is essentially a vision science experiment. The five elements need not be considered in the exact order presented here, but all should ideally be present to some extent. The particular issues examined can be driven by theoretical considerations, by challenges encountered by visualization researchers, or simply be the result of curiosity about how a particular visualization system operates. In any event, the behavioral regularities and mechanisms uncovered by such experiments—along with the new kinds of questions inspired by such investigations—form the essence of how visualization can contribute to vision science.

To give a sense of how this approach works and what it can accomplish, various representative studies are reviewed below. The focus is on the two main domains of visualization, the creation of static graphical representations and the control of interactive operations (Buja, Cook, & Swayne, 1996; Roth, 2012), both of which have correlates in human perception. These examples will hopefully make clear how this approach can be used to investigate our visual intelligence, as well as the kinds of contributions that the study of visualization may make towards a better understanding of vision.

## Graphical representations

###  

Given that the investigation of visualizations can help us to better understand human vision, what form might such assistance take? Understanding a given visualization is more of a challenge than it might at first seem: relatively little is known about the perceptual and cognitive mechanisms involved. First-wave work on visualization systematized the basic elements of graphical representation, and compared performance for designs such as scatterplots, line graphs, and pie charts (e.g., Bertin, 2010/1967; Cleveland & McGill, 1984, Lewandowsky & Spence, 1989). But although such studies were valuable, they also had limitations—for example, they did not always use effective methodologies and rarely connected their results to perceptual mechanisms (see Kosara, 2016; Kosara & Haroz, 2018). And although such work continued well past the initial wave of activity (e.g., Boynton, 2000; Doherty, Anderson, Angott, & Klopfer, 2007; Meyer, Taieb, & Flascher, 1997), it did not flourish as much as one might have hoped. In particular, it did relatively little to advance our understanding of visual perception.

The newer approach, in contrast, considers graphical representations as stimuli amenable to controlled, systematic experimentation. Minimal versions are generally used (e.g., with simple lines as axes and no labels), with well-defined experimental tasks based directly on the graphical properties present (e.g., average size or color). Semantic knowledge need not be—and usually is not—involved. This strategy not only decreases the likelihood of extraneous factors affecting the results, but also improves the likelihood that these results can be directly linked to perceptual mechanisms.

There are, of course, reasons why this approach might not succeed. One is that observers may vary in their responses to such a degree that well-defined regularities in perception do not exist, or at least, cannot be reliably measured. Another is that, even if such regularities exist, they might not connect to a well-defined set of mechanisms: performance may result from several different mechanisms interacting in complex ways. Finally, the effects of expertise could muddy the separation between general lower level perceptual abilities and higher level individual skills.

Such concerns, however, have not always materialized. For example, although effects of expertise do appear in some tasks—for example, experts can distinguish between different populations of items in a scatterplot more effectively than can novices (Lewandowsky & Spence, 1989)—they are not encountered in others (e.g., Harrison, Yang, Franconeri, & Chang, 2014; Meyer et al., 1997), suggesting that many tasks have a basic stage carried out by relatively low-level processes that are not highly dependent on training (e.g., see Rensink, 2017). Moreover, as the following examples show, interesting and occasionally counterintuitive behavior can be encountered in many situations, which can yield insights into visual perception that may not have been possible—or at least, not uncovered as quickly—by investigating more traditional concerns of vision science.

### Magnitude in bar charts

**Figure 2.**
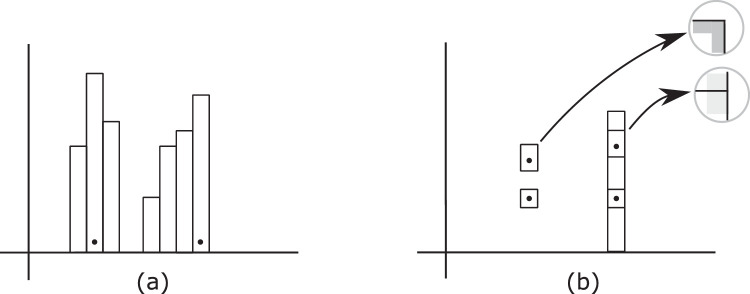
Example stimuli for perception of magnitude in bar charts. Participants are shown a set of bars (some marked by a dot), and asked to estimate the percentage by which the larger of the two marked bars exceeds the other. (a) Separation effect. Estimates of relative height are less accurate when bars are separated than when adjacent. (b) Stacking effect. Estimates for isolated components (left) are less accurate when these are incorporated into a stacked set (right). This decrease in accuracy suggests that L-junctions are interpreted as corners of a distinct object, but when they become T-junctions, the stems are interpreted as marking segments of the same object.

Bar charts are a popular way to simultaneously show magnitude in several categories, such as the amount of rainfall at various locations. However, relatively little is known about how they work. Building on the earlier work of Cleveland and McGill (1984) and Simkin and Hastie (1987), Talbot, Setlur, and Anand (2014) showed participants bar charts that were simplified in several ways (e.g., no scales on the axes; only one type of bar), and gave them just one task: estimate the difference in height between the two bars marked by dots. Performance was then measured as a function of the configuration of the bars (Figure 2a). Results showed several interesting patterns. To begin with, perception of relative height is easier and more accurate when the target bars are immediately adjacent to each other; this separation effect is largely independent of the presence of other bars (distractors) in the vicinity. Talbot et al. also confirmed that performance is markedly better when the bottoms of the bars are aligned, indicating that length per se is not the relevant variable. Rather, the relevant variable for estimates of individual bars appears to be the *difference* in the positions of the tops of the bars; this likely connects to the use of spatial relations in position (Nothelfer & Franconeri, 2020). For judgements of the average length of a set of bars, the relevant variable does seem to be length, but is the *sum* of the lengths (or areas) rather than their average value (Yuan, Haroz, & Fraonconeri, 2019).

Talbot et al. also found that the presence of distractors taller than the target bars tends to degrade the estimation of the magnitude of individual bars, regardless of the location of the targets. Because the separation of the targets does not matter, performance cannot be based on a simple comparison of the tops of the bars. Estimation also seems to be affected by the presence of noisy data between the target bars (Zacks, Levy, Tversky, & Schiano, 1998). This effect—and perhaps the separation effect as well—may ultimately be due to the interference caused by grouping or crowding in peripheral vision (e.g., Keshvari & Rosenholtz, 2016; Rosenholtz, 2011). This issue worth is exploring further.

The perception of relative magnitude was also found to be poor for components that are incorporated into a stacked set (Figure 2b). One possible explanation is that stacking causes the L-junctions delineating each component to become T-junctions, with the stems of these T-junctions then being unable to maintain their interpretation as boundaries of components of distinct size (Talbot et al., 2014). If so, this proposal would be in accord with the conclusions of other studies as to how T- and L-junctions affect the way that simple figures are perceived (e.g., Adelson, 2000; Enns & Rensink, 1991), and possibly draw on the same perceptual mechanisms. One way of exploring this further might be via the effects of perceived complexity, which seems to slow down the comprehension of bar charts, with 1.7 seconds added for each component that is perceived (Fischer, 2000; Hollands & Spence, 1998).

### Proportion in pie charts

Pie charts have long been used in visualization, but again, little is known about how they work. To investigate, several studies focused on the most common function of pie charts: representing proportion. Simplified charts were used that had no labelling, with the task being to estimate the proportion of a single target sector, or “slice,” relative to the whole (Kosara & Skau, 2016; Skau & Kosara, 2016). Observers were tested on variants such as pie charts with their centers removed, or with slices not aligned with the periphery; performance was measured as a function of the angle subtended by the slice. Results showed that—contrary to traditional belief (e.g., Simkin & Hastie, 1987)—the critical variable is not the angle of the slice; rather, it seems to be either the area of the slice or the length of the arc along its periphery (Kosara & Skau, 2016; Skau & Kosara, 2016). Subsequent experiments using pie charts depicted at various angles of viewing—which leaves relative areas unaffected, but not angles or arcs—indicated that the relevant quantity is likely area (Kosara, 2019).

These results in turn raise the issue of how area is perceived in a given shape. Chong and Treisman (2003) found that the average perceived size of a set of briefly viewed circles is best described by a value between the means of the areas and the diameters, suggesting that area was not directly perceived by at least some participants. In addition, Morgan (2005) and Nachmias (2008) found that the area of rectangles and ellipses seems to be assessed via two single-dimensional measurements—essentially, width and height—with no high-accuracy perception of area itself. Extending such considerations to the perception of other shapes (such as those of pie chart slices) would be an interesting direction for future work; the perception of proportion could be an important part of this process.

### Use of color

Color is a common way to distinguish different categories of data in a display. Although the perception of color has received considerable attention in vision science, research has generally focused on elements subtending at least a few degrees of visual angle, or on patterns that vary at the pixel level (e.g., Poirson & Wandell, 1993). To explore what happens in graphical representations, Stone, Szafir, and Setlur (2014) asked participants to judge whether two adjacent squares (of a size typical of visualization applications) were the same color or not; the difference in color between the squares was adjusted until a JND was reached. Results showed an important regularity: color JND was not constant, but rather, was a linear function of the inverse size of the elements tested (Stone et al., 2014; Szafir, 2017). The underlying mechanism is not known, but may involve the intraocular scattering of light in the retina (Carter & Silverstein, 2010). Interestingly, color perception at these scales also seems to be influenced by shape (Smart & Szafir, 2019).

Another use of color is for attentional selection. Whereas items can be readily selected based on color for virtually all tasks (Wolfe & Horowitz, 2004), such selection seems to fail when estimating correlation in scatterplots. When a second (distractor) set of dots of lower correlation is added, JNDs for the target set always increase, regardless of the size of the color difference (Elliott & Rensink, 2019, 2020). In contrast, when the same stimuli (including colors) are used in estimating the number of items or average position of a given subset, such selection can be done quite easily (Chong & Treisman, 2003, 2005; Elliott & Rensink, 2019). This task dependence of attentional selectivity is a phenomenon unlikely to have been uncovered by investigating more traditional issues.

### Redundant encoding

A common belief in visualization is that multiple classes in a display can be more easily distinguished if they differ in several feature dimensions rather than just one—for example, if items differ in both shape and color, rather than shape or color alone. To determine if redundant encoding of this kind actually does help, Nothelfer, Gleicher, and Franconeri (2017) showed participants simple displays containing dozens of small items of various shapes and colors. One of the display quadrants lacked elements of a particular color, shape, or particular color and shape conjunction; participants were asked to report the location of this quadrant. Performance was best when the target quadrant lacked items differing in both dimensions from the items in the other quadrants. This finding suggests that multiple features can be concurrently selected when items receive only diffuse attention, generalizing what is known for individual objects that are given focused attention (Egeth & Pachella, 1969).

### Average value in scatterplots

Scatterplots have long been used to support the perception of various kinds of structure in a dataset (e.g., see Friendly & Denis, 2005). One of their most common uses has been to enable the visual estimation of average value (i.e., average height of a set of dots). The average position—as well as other features—of a set of dots can be rapidly ascertained via ensemble perception (e.g., Alvarez & Oliva, 2008), suggesting that this may underlie the perception of average values in scatterplots (Szafir et al., 2016). This possibility was examined by Gleicher, Correll, Nothelfer, and Franconeri (2013), who showed observers simple displays containing two sets of dots, with the dots in each set having distinctive colors and shapes; observers were then asked to detect differences in the average heights of the two sets. Consistent with known characteristics of ensemble perception (Alvarez & Oliva, 2008; Chong & Treisman, 2003), performance was found to be invariant across different numbers of dots (15–75), and was best when the two sets had large differences in color.

Curiously, redundant encoding—using both color and shape—had no effect on the perception of average values, even when displays were presented for as long as 10 seconds (Gleicher et al., 2013). This outcome differs from the findings of Nothelfer et al. (2017), who—as described elsewhere in this article—did find redundancy effects when the task was to localize a texture quadrant. This difference may indicate that the ability to select multiple dimensions is somehow task specific, perhaps having to do with the noise resistance of the average estimation process (Nothelfer et al., 2017); alternatively, redundancy may only benefit processes that operate over brief time scales (see Szafir et al., 2016).

### Correlation in scatterplots

**Figure 3.**
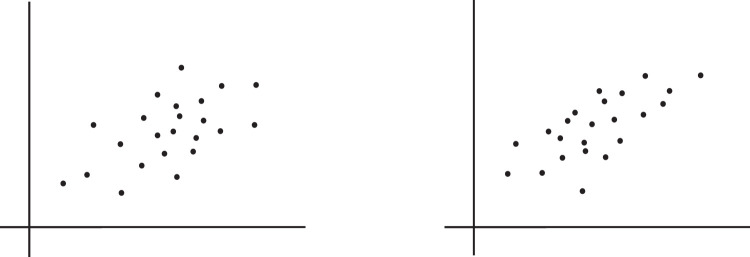
Example stimuli for discrimination of correlation in scatterplots. Two side-by-side scatterplots are presented, with participants asked to select the one appearing to be more correlated. The averages and marginal distributions of the distributions of dots in both directions are equated, so that correlation is the only property that differs between the two plots. These differences are adjusted until performance reaches some predefined threshold, for example, 75% correct, with the corresponding difference then taken as the JND.

Rensink and Baldridge (2010) investigated the perception of Pearson correlation in simple versions of scatterplots that contained no ticks or labels along the axes (Figure 3). Two aspects of performance were examined: precision and accuracy. Precision was measured in the same way as for, say, size, or color: the minimum difference needed to notice that one of two side-by-side scatterplots has a higher correlation (Figure 3). Estimated magnitude was assessed via a bisection technique similar to that used for assessing the perceived magnitude of properties such as lightness (Plateau, 1872), that is, adjusting a middle plot so that its apparent correlation is midway between those of two reference plots.

Results showed that, for a wide range of designs and correlation values, performance can be described by two general laws: a linear Weber-like law for JND (which decreases as correlation increases) and a logarithmic Fechner-like law for magnitude (Rensink, 2014; 2017). Given the pervasiveness of Weber's law in visual psychophysics (e.g., see Pardo-Vasquez et al., 2019; Ross, 1997), the finding of such laws here suggests the involvement of a perceptual process rather than high-level cognition. Indeed, Li, Martens, and van Wijk (2010) found that the process involved approaches the theoretical optimum possible, at least when sample sizes are small. Importantly, discrimination and estimation appear to be linked, with the intercept of the JND line corresponding with the amount of bend in the logarithmic curve (Rensink & Baldridge, 2010). This linkage suggests that the Fechner assumption holds—that is, that each JND corresponds with an equal perceptual step (Rensink, 2017). Such a phenomenon is not generally encountered for continuous sensory properties such as size or brightness, where perceived magnitude is a power function of the corresponding physical magnitude. This finding suggests that the pattern of behavior found for correlation may be a signature of the perception of abstract numerical quantities, that is, quantities with no linkages to any sensory quality. The involvement of such quantities might also explain why subsets of dots cannot be selected on the basis of color (Elliott & Rensink (2019)—the sensory information may simply not be there.

As to mechanism, one possibility consistent with these behaviors is *entropy theory*: observers infer the shape of a probability density function associated with the dot cloud, and use the logarithm of its width—corresponding with the information entropy (or disorder) of the dot cloud—as a perceptual proxy for correlation (Rensink, 2017). Alternate explanations such as the size of the bounding box of the dots can be ruled out, as can measures such as average distance of the dots to the regression line, leaving density functions as the most likely way to account for the results (Elliott & Rensink, 2020; Yang et al., 2019). Moreover, this account implies that the Fechner assumption—that each JND represents an equal perceptual difference—has the interesting form that each JND represents an equal number of bits (Rensink, 2017).

Entropy theory asserts that the relevant quantity for correlation perception in scatterplots is the width of an inferred probability density function, rather than its first- or second-order moments (e.g., averages or standard deviations). As such, work on correlation perception may be a complementary way to explore mechanisms related to those that underlie ensemble perception. The abstract nature of density functions may also account for the finding of general Weber-like laws for other graphical representations, whose appearances can differ considerably from those of scatterplots (Harrison et al., 2014; Rensink, 2014). In any event, such discoveries would not likely have been found as readily using more traditional approaches.

## Interaction in visualization

###  

Although the power of visualization has historically been based on static graphical representations, advances in technology have enabled an extension to more dynamic displays, usually under interactive control (Pike, Stasko, Chang, & O'Connell, 2009; Yi, Kang, Stasko, & Jacko, 2007). Such interaction—for example, moving through various subsets of data, spotting an outlier in one of them, then drilling down to get more information about it—often plays a critical role in visualization, especially when a task is incompletely defined or a dataset poorly understood (Fekete, van Wijk, Stasko, & North, 2008; Roth, 2012; Thomas & Cook, 2005). Given that visual perception itself is also inherently interactive (e.g., Findlay & Gilchrist, 2003; Land & Tatler, 2009; Rensink, 2000), the question then is what can be learned about vision—and in particular, the interdependence of lower level perception and higher level cognition—by considering the corresponding aspects of visualization.

Early investigations of how visualizations assist in complex tasks tended to focus on their cognitive aspects, for example, the conceptual schemas underlying the comprehension of a given graph (e.g., Pinker, 1990; Shah, 2002; Wickens & Flach, 1988). But such work had relatively little impact on our understanding of vision. A more productive approach for purposes here may therefore be to focus instead on the control of processes that enable the discovery of structure in data independent of semantics, and in particular, on processes that enable the *interaction* of perception and cognition.^2^

It is worth noting that many of the low-level “semantics-free” operations in visualization have recognizable correlates in human perception—for example, identification, localization, clustering, grouping, linking, panning, zooming, and filtering (Amar, Eagan, & Stasko, 2005; Roth, 2012). Indeed, selection is sometimes described as a component of these operations in the same way that visual attention is sometimes described as the selective application of various operations (e.g., Rensink, 2015). Such considerations suggest a degree of isomorphism between vision and visualization, so that investigating the interactive aspects of one may help us understand those in the other.

**Figure 4.**
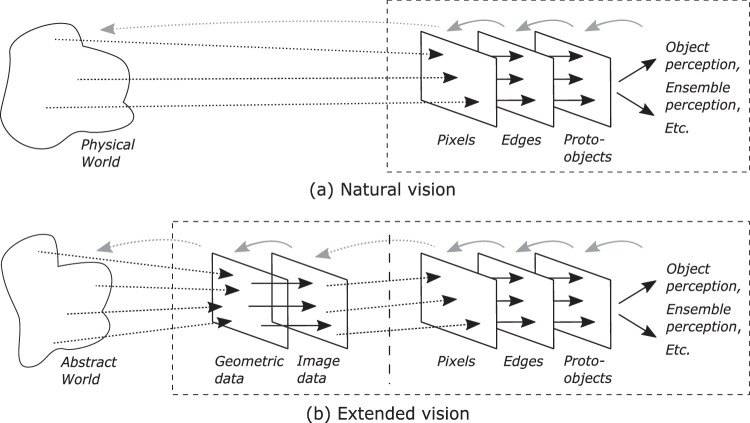
Extended vision thesis. (a) Schematic of natural vision. Structure of the physical world is estimated via a set of processes that use both high-level knowledge and low-level data. Feedback is present at all levels; it is applied to the physical world via actions such as eye or hand movements. (b) Schematic of extended vision. Here, structure in a dataset is perceived via a set of processes that use both high-level knowledge and low-level input, with visual input to the user now being an intermediate stage of processing, taking its input from the graphical representations formed by the visualization pipeline. Feedback is still present at all levels, with analyst commands controlling the visualization pipeline, including selection of data. (Based on Rensink, 2014).

One characterization of this isomorphism is what might be called the *extended* *vision thesis*: the informational linkages between human and machine create a composite system that can perceive structure in an abstract dataset in much the same way that a natural visual system can perceive structure in the physical world (Figure 4). In this view, the visualization pipeline that maps abstract data to a graphical representation (Card et al., 1999) is essentially an extension of low-level vision, enabling the human analyst to perceive structure in information from sources other than physical ones (Rensink, 2014). An interactive system adds feedback control; this exists at all stages, resulting in a rough architectural consistency with what is found in the human visual system. The resulting process is often divided into a lower level loop that extracts data from the input image and feeds it to a higher level “sensemaking loop” that in turn controls the lower level loop (Pirolli & Card, 2005). This process has clear similarities with the “cycle of perception” in computer vision (Mackworth, 1978) and the “perceptual cycle” in human vision (Neisser, 1976), in which perception is influenced by high-level knowledge, which in turn is influenced by perception. A popular mantra for exploratory visualization is “overview first, zoom and filter, then details on demand” (Shneiderman, 1996); this has an echo in visual perception, where a global view of the scene can guide subsequent attentional filtering, construction, and tracking of items (e.g., Rensink, 2000). For vision and visualization, then, many of the problems and solutions may be much the same.

As for the case of graphical representations, there are no guarantees this approach will necessarily be productive. For example, the kinds of tasks carried out interactively may simply not be amenable to well-defined solutions, or, if there are such solutions, it may not be possible to implement them using operations available in current visualization systems. And even if this could be done, the unnaturalness of the stimuli and task—not to mention the technology itself—might result in strategies and operations that differ considerably from those used in natural vision. And finally, even if an isomorphism of this kind does exist, the approach described here may be too reductive—the simplifications and controls needed to carry out experiments of the kind advocated here might not be able to isolate the key components used in more realistic situations.

Although these concerns are valid in theory, they need not be so in practice. For example, the naturalness of a display does not seem to be critical for performance on interactive tasks (Smallman & St. John, 2005; Hegarty, Smallman, & Stull, 2012), suggesting that simplified, controlled experiments may often succeed at capturing important aspects of interaction. Moreover, any limits encountered that are not due to the machine component (such those involving timing or memory) can reliably be attributed to the human system. And finally, as the following examples show, there is also empirical evidence to believe that interactive visualization can often provide an interesting way to explore how lower level processes interact with higher level ones, and how these together interact with the world.

### Latency of operations

A common recommendation for interaction is that the latency of operations—the time between motor command and updated display—should be less than about 300 ms if performance is to be unaffected, and experienced as seamless (Ballard, Hayhoe, Pook & Rao, 1997; Gray & Fu, 2004; Vozar & Tilbury, 2014). As a step toward understanding this phenomenon, Liu and Heer (2014) examined the effect of latency on exploratory visual analysis, using a simplified version of a real-world dataset. Participants were given a small set of simple operations (e.g., pan and zoom) and asked to find interesting patterns, such as outliers and data abnormalities, and to suggest hypotheses to account for them. Performance was assessed in terms of the number of observations and hypotheses made, and measured as a function of the latency of the commands.

The latency of the basic operations was typical of most visualization applications, that is, 100 ms or so, and this supported behavior typical of this kind of visualization system. Interestingly, adding a 500-ms delay to each operation led to fewer hypotheses and poorer exploration strategies; adding a 1000-ms delay made the system unusable. These results suggest that optimal sequences of operations may decay to less optimal ones—and even disintegrate entirely—if not carried out sufficiently quickly.

Interestingly, participants initially tested on high-latency conditions did not improve in subsequent low-latency ones, pointing to a degree of hysteresis: a reluctance to change strategy once a sequence of operations had been set up (Liu & Heer, 2014). The nature and extent of such hysteresis would be worth exploring. Another possible extension would be to map out the latency limits even when commands are not involved (when watching slow-moving animations, say); investigations of this kind might provide a useful way to map out the dynamics of the processes involved in a perceptual task.

### Role of motor control

Visualizations often use interaction to facilitate perception of the three-dimensional (3D) structure of a particular dataset or world (Buja et al, 1996). An interesting issue is the extent to which the resulting percept depends upon active motor control: does it require the motor *action* itself, the *intent*, or perhaps just the *result*?

Keehner et al. (2008) examined this issue by comparing the perception of 3D structure for rendered objects displayed interactively (i.e., rotated and translated via user commands) against those displayed via an animated sequence of two-dimensional images. The ability of observers to determine the 3D structure of each object was assessed via their accuracy in determining the cross-section of the object for an arbitrary cutting plane. Results showed that when an object was displayed using an optimal sequence of views—where each view contained the maximum amount of relevant information—the ability to infer cross-sections was unaffected by whether the object was perceived actively (i.e., via user commands) or passively (i.e., viewed as an animated sequence of images). This finding suggests that performance is determined entirely by the information displayed, with active control important only to the extent that it makes task-relevant information available. How users eventually learn the optimal—or at least, near-optimal—sequence of views for a given object remains an open issue.

The results of such studies may connect with work in vision science on the information needed to recover 3D structure from motion sequences (see Anderson & Bradley, 1998).

They may also connect with the more general issue of the role of prediction in perception (e.g., see Cavanagh, 1997; Hawkins & Blakeslee, 2004): could the predictions enabled by the intent of a motor command somehow facilitate at least some perceptual processing (cf., efferent copy in eye movements)?

### Selection of operations

Another important issue in perception is the selection and arrangement of a set of operations for a given task when different sequences can yield results that are largely similar. This prospect was examined by Gray and Fu (2004), who investigated whether users preferred functionally equivalent operations that were internal or external to the viewer (e.g., mental rotation vs. rotating an image in the display). They asked users to program a device using a visual display covered to varying extents for different amounts of time; the extent to which internal operations (involving memory) were used in preference to external ones (involving fixation of the display) was assessed via eye movements and the amount of information accessed.

Results, which were fairly similar across users, were consistent with a “least-effort” principle, which asserts that if a given task can be carried out by several possible sequences of operations, the one selected—at least for operations at the 300-ms scale—is that which minimizes the overall time needed, regardless of the mechanisms involved. A similar experiment examining the manipulation of blocks on screen to create a copy of a given pattern gave much the same results (Gray, Sims, Fu, & Schoelles, 2006). To the extent that this principle holds in general (and for other time scales), it would imply that—apart from timing—the physical nature of interaction operations in a perceptual task is largely irrelevant. If so, this principle could have important applications to natural as well as extended vision.

One example of this is might be foraging, where all the relevant targets in a display (or part of a display) must be examined sequentially (Thornton & Horowitz, 2015; Wolfe, 2013). Strategies for this task seem to be largely if not entirely determined by temporal constraints, with little effect of response modality (Thornton, de'Sperati, & Kristjánsson, 2019). Indeed, it has been suggested that the least-effort principle may explain the switches in attentional strategies that occur when foraging is carried out at different speeds, for example, when the marking of individual items is synchronized to the beat of a metronome that runs at various rates (Thornton, Nguyen, & Kristjánsson, 2020).

## Future directions

The work reviewed here provides some grounds for believing that the investigation of visualization can be an effective way to explore various aspects of visual perception, resulting new kinds of issues, new aspects of behavior, and new kinds of possible mechanisms and principles. These examples are not intended to constitute an exhaustive survey of what can be done using this approach; rather, they are intended to provide a sense of the kinds of issues that can arise, the ways these might be investigated, and the kinds of findings that might result. Many aspects of visualization—and their explanations—remain to be explored.

### Graphical representations

Although the investigation of graphical representations is undoubtedly the subdomain of visualization that has received the greatest attention, much can still be done. To begin with, investigation could be extended into several of the results already obtained. Examples of relevant questions include the following:

• Why does the estimation of average magnitude for sets of bars in bar charts involve the sums of bar lengths or areas (Yuan et al., 2019)? How does this relate to ensemble coding, which—at least for area—involves averages rather than cumulative values (Raidvee et al., 2020)?
• Might other kinds of geometric relations exist beyond those uncovered by Nothelfer and Franconeri (2020)? And what other summary statistics might be determined by ensemble processes beyond mean and range or variance (Szafir et al., 2016)?
• What is the nature of the perceptual representations that underlie correlation perception? Are the Weber- and Fechner-like laws found for various graphical representations (Harrison et al., 2014; Rensink, 2014) more general yet? And does the finding that attentional selection fails for correlation estimation in multiclass scatterplots (Elliott & Rensink, 2019) but not for estimation of averages (Chong & Treisman, 2003, 2005) imply the existence of different forms of ensemble coding?

**Figure 5.**
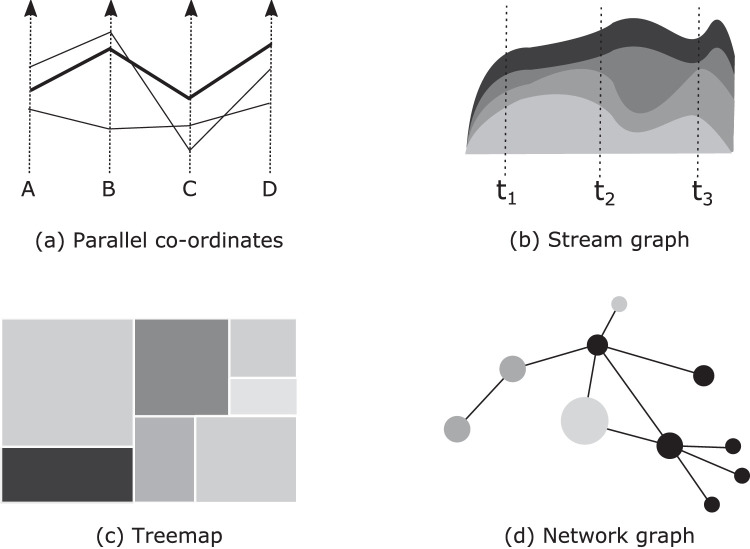
Examples of designs for graphical representation. (a) Parallel coordinates. Each data element is a line, with its intersection with a co-ordinate axis representing the value for that dimension. (b) Stream graph. This is a time series, where the thickness of each stream represents its value. (c) Treemap. Here, each rectangle represents the relative quantity for a set of categories. (d) Network graph. Nodes represent entities, with links representing the associations between them.

Note that investigation need not be limited to the graphical representations already discussed. Hundreds of different representations are currently in use (Harris, 1999), with various appearances and supporting various functions. (For examples, see Figure 5.) For most, the perceptual mechanisms involved are largely unknown, as is the explanation of various interesting behaviors. For example, Kong, Heer and Argawala (2010) found that people distinguish values in a treemap (Figure 5c) best when the components are rectangles with diverse aspect ratios; curiously, squares are not easy to compare to each other, nor can rectangles be easily compared if they have extreme aspect ratios.

Investigation is also just beginning of other aspects of perception, such as the effect of graphic design on memory (Borkin et al., 2013), the representation of uncertainty in data (Bonneau et al., 2014), and the number of data dimensions that can be effectively displayed in a single image (Halford, Baker, McCredden, & Bain., 2005; Healey & Enns, 2012). As such, it seems that the study of graphical representations can offer a large number of ways to investigate various aspects of visual cognition (see also Kosara, 2016); indeed, it is difficult to imagine a domain better suited for this kind of work.

### Interaction in visualization

Similar to the case of graphical representations, investigations could also be extended to uncover more about the mechanisms involved in interaction. Questions could include the following:

• How does latency affect visual processing? Is there a single value such that all processes in a sequence remain unaffected if latency stays within that limit? Work on teleoperation—for example, Vozar & Tilbury, 2014—may be of relevance here. And given that the timecourse of conscious cognition differs from that of automatic actions (e.g., Milner & Goodale, 1995), could this approach also help to elucidate the extent to which consciousness is involved?
• To what extent are interaction commands necessary for perception in a dynamic display? In particular, to what extent is the information in the commands themselves—for motor actions or otherwise—needed to perceive various kinds of structure? What kind of information is needed at what time?
• How general is the least-effort principle for selecting operations in perceptual sequences? Foraging strategies for multiple-target search in simple environments seem to be consistent with this principle (Thornton, Nguyen, & Kristjánsson, 2020). Does it also apply to foraging more broadly construed (Pirolli & Card, 2005; Pirolli, 2007), where information is accumulated over time for various tasks?

There is also potential in other operations. For example, comparison is not only an important part of perception (Farell, 1985), but also of visualization (Roth, 2012). Although there exists a diverse array of designs that support comparison in visualization, all seem to rely on three basic elements: juxtaposition (side by side), superposition, and explicit encoding of differences (Gleicher et al., 2011); these elements may reflect distinct levels of information integration. There may also be lessons here in regards issues such as the encodings used in working memory.

Many other open-ended tasks might also be studied by the use of simplified versions in simple environments (e.g., see Prpic et al., 2019; Thornton, de'Sperati, & Kristjánsson, 2019). Consider, for example, navigation. In visualization, this process is often facilitated by the use of an overview map (e.g., Beard & Walker, 1990); could its counterpart in perception be the coarse representation of the immediate environment sometimes thought to guide the perception of scenes (Rensink, 2000)? Could we discover why such maps facilitate performance, and have this help us to understand how scenes are perceived? Related issues are why particular kinds of landmarks prove useful (e.g., Parush & Berman, 2004; Vinson, 1999), and the extent to which navigation is possible for spaces of higher dimension (e.g., Buja et al., 1996); results here could cast additional light on the extent to which our visual systems are hard-wired for the world in which we live. Also of interest would be the way that navigation is carried out on small screen devices, where different strategies are likely used (e.g., Burigat, Chittaro, & Gabrielli, 2008). This approach might even extend to issues such as the kinds of physics that can exist—or at least, be perceived—in such environments (Ullman, Spelke, Battaglia, & Tenenbaum, 2017).

### Other aspects

Visualization is a complex area, standing at the intersection of several research traditions (Munzner, 2014; Spence, 2014). Some of the issues involved (e.g., efficient algorithms for data transformation) are unlikely to ever concern vision science. Others (e.g., workflows for data analysis) involve cognitive mechanisms that are incompletely understood at the moment, and may never affect our understanding of visual perception. And yet others (e.g., design for a particular application) may involve vision, but the prevalence of subjective and context-specific factors may prevent them from forming a part of systematic considerations (Rensink, 2014).

Even granted this, however, other aspects of visualization could still help us better understand perception and its relation to cognition. Examples include the following:

• What is the basis for thinking about abstract categories in terms of geometric or topological spaces (e.g., see Gärdenfors, 2004)? An isomorphism of some kind with perceived space is evidently involved. But how is this isomorphism established, and how much structure can it contain? More generally, what is the basis of effective visual metaphor (Paley, 2009; Ziemkiewicz & Kosara, 2008)?
• What is the structure of a visual task? Although work has been done on how vision is used in everyday life (e.g., Gibson, 1966; Land & Tatler, 2009), relatively little is still known about the structure of complex visual tasks. In contrast, considerable work in visualization has been done on abstract task taxonomies and the decomposition of tasks into simpler operations (e.g., see Amar, Eagan, & Stasko, 2005; Roth, 2012; Schulz, Nocke, Heitzler, & Schumann, 2013). To the extent that the extended vision thesis holds, such work may reflect something about the nature of the tasks carried out in natural vision.
• To what degree can visual representations incorporate—or at least support—semantics? Early work (e.g., Pinker, 1990) focused on how graphs can help in understanding a situation. Other kinds of visualization could also provide useful perspectives here—for example, the understanding of maps (MacEachren, 1995) and diagrams (Massironi, 2010; Tversky, 2011).

Studies along some of these lines have already begun. For example, Lin et al (2013) investigated the issue of semantic associations with color (e.g., blue and oceans), comparing performance on barcharts where colors corresponded—or did not correspond—with the categories depicted. Results showed a small but significant speedup when semantic associations were consistent; these may connect to the color-concept associations found in visual search (Treisman & Souther, 1985). Schloss et al. (2019) found that associations can also exist between color and quantity, although these do not seem to exist between color and emotional valence (Schloss, Witzel, & Lai, 2020). Work of this kind might also help to clarify not only the relationship between the functional aspects of perception and cognition, but also between these and aesthetics (e.g., Filonik & Baur, 2009).

## Summary

It has been argued here that the interaction of visualization and vision science can be expanded to include the controlled investigation of visualization itself. Such studies can suggest new kinds of questions, uncover interesting new phenomena in perception, and accelerate the uncovering of various perceptual mechanisms. Visualization is a domain well-suited for the exploration of our visual intelligence, with widely used design elements likely embodying considerable information not only about the nature of the perceptual processes involved, but also their links with higher level cognition. Studies of visualizations have accordingly raised new questions about vision, such as our ability to perceive abstract structure (rapidly or otherwise) in an image, and the degree to which our perceptual system is hardwired for the natural world. They have also led to the discovery of behaviors, such as the general laws of correlation perception, unexpected failures of attentional selection, and a possible signature of abstract information; they have also led to the proposal of mechanisms such as entropy estimation, as well as a principle for selecting the operations to be used in a perceptual sequence. Such studies therefore not only challenge several existing views about vision (e.g., the nature of attentional selection), but have also extended our knowledge of perception in new directions.

Based on this information, it might be worth viewing visualization not only as an important area *for* vision science, but also as an important area *of* vision science, analogous to, say, scene perception or material perception, which were developed based on a recognition of the shortcomings of existing techniques and stimuli. Indeed, the investigation of visualization might not only extend our knowledge of perception, but might also provide a theoretical grounding for several aspects of visualization itself (Rensink, 2014). In any event, the usefulness of a two-way connection between vision science and visualization would seem to be clear. Together with the “forward” connections that have already been developed, reverse connections of the kind described here can be the basis of a productive feedback loop in which developments in each area can spur developments in the other, resulting in a body of seamless interdisciplinary work created by—and of benefit to—researchers in both fields.

## Footnotes

1 A closely-related related area is *scientific visualization*, which is similar but focuses on the kinds of information particular to the natural sciences. Other related fields include *statistical graphics* (the visualization of statistical data), *cartography* (the visualization of geographic data) and *visual analytics* (the use of visualization for sophisticated analytical reasoning; its exploration aspect is sometimes referred to as *visual data mining*). “Visualization” is used here as a cover term for all of these.

2 The division between perceptual and conceptual is broadly similar to that between low- and high-level interaction, which involve interface and problem-space operations respectively (Pike et al., 2009). Higher-level operations often depend on factors such as user characteristics (e.g., expertise), context (e.g., motivation), and the particulars of the task, which are difficult to take into account; as such, they will not be further discussed here.
